# Non-invasive computer navigation can quantify the pivot shift maneuver with good to excellent reliability in healthy volunteers

**DOI:** 10.1186/s40634-020-00239-5

**Published:** 2020-04-17

**Authors:** Edoardo Monaco, Giorgio Bruni, Sara Lo Torto, Alessandro Carrozzo, Matthew Daggett, Alessandro Annibaldi, Adnan Saithna, Andrea Ferretti

**Affiliations:** 1 Orthopaedic Unit and Kirk Kilgour Sports Injury Centre, S. Andrea Hospital, University of Rome La Sapienza, Rome, Italy; 2 Kansas City University of Medicine and Biosciences, Kansas City, MO USA

**Keywords:** Navigation system, Surface marker, Inter- and intra-observer reliability, Rotatory laxity, Physical examination, Pivot shift, Knee, Ligaments

## Abstract

**Purpose:**

The aim of this study was to determine the inter- and intra-observer reliability of knee laxity assessment using a non-invasive navigation system in a population of healthy young athletes. It was hypothesized that knee laxity parameters recorded using non-invasive computer navigation would demonstrate good inter- and intra-observer reliability.

**Methods:**

Healthy volunteers aged between 18 to 30 years were recruited to the study. Static and dynamic knee laxity parameters including anterior tibial translation and tibial rotation during the pivot shift test were recorded on awake patients using non-invasive computer navigation by two independent observers: at the first visit each athlete was evaluated by the consultant and resident surgeons independently; 6 weeks after the first visit all the participants were re-tested only by the resident surgeon. Inter- and intra-observer reliability was calculated and then interpreted according to Cicchetti’s criteria.

**Results:**

One hundred healthy volunteers were recruited to the study, of these 38 were women (38%), and the average age was 25.5 ± 2.4 years. According to Cicchetti’s criteria the intra- and inter-observer reliability for static measurements were fair for anterior tibial translation (0.572 and 0.529, respectively) and excellent for total passive tibial rotation (0.859 and 0.883, respectively). For the dynamic measurements of translation and rotation during the pivot shift maneuver both measurements demonstrated good to excellent reliability with intra and inter observer reliability ranging from 0.684 to 0.936.

**Conclusion:**

Non-invasive navigation for the assessment of knee laxity is associated with fair to excellent inter- and intra-observer reliability in a population of healthy volunteers.

## Background

The Lachman and Pivot Shift (PS) tests are respectively recognized as the most sensitive and specific physical examination tests for the diagnosis of anterior cruciate ligament (ACL) rupture [1, 15] . However, these tests, particularly the PS, have large subjective components to them and this limits their inter- and intra-observer reliability. *Noyes* et al. [25] reported that grading of the PS test is inaccurate and irreproducible and suggested that the use of the PS should be viewed with caution. Despite this, the PS test is reported in the majority of studies evaluating ACL-injured patients. However, considerable effort has been made to try and reduce some of the subjective aspects of these tests. This has included a preference to perform them under anesthesia (to minimize the effect of guarding and apprehension), using standardized examination techniques, and also utilizing technology to try and avoid the requirement for the observer to estimate tibial translation and rotation. This technology has included the use of electromagnetic sensors [5, 12, 13, 22], accelerometers [10, 18, 20], image analysis [11, 23] and navigation systems [14, 17], all of which have been developed and evaluated for their role in reducing subjectivity and improving inter- and intra-observer reliability in the assessment of knee laxity parameters. Computer-assisted navigation offers a unique advantage over the other technologies mentioned, in that it is able to precisely quantify motion with 6 degrees of freedom (DoF) [19]*.* However, traditional navigation systems are not frequently used to assess knee laxity parameters due to their invasive nature and the requirement to affix the transmitter attachment to bone using metal pins. For that reason, the use of non-invasive navigation systems has been considered as an alternative.

The aim of this study was to determine the inter- and intra-observer reliability of knee laxity assessment using a modified OrthoPilot ACL navigation system, with non-invasive skin markers, in a population of healthy young athletes. It was hypothesized that knee laxity parameters recorded using non-invasive computer navigation would demonstrate good inter- and intra-observer reliability.

## Methods

Institutional review board approval was granted for this study (IRB number blinded for journal review). Prospective recruitment of healthy athletes aged between 18 and 30 years was undertaken between January and August 2019. Invitations to participate in the study were sent to the coaching staff and all team members of sports teams (running, soccer, volleyball, rugby, and tennis) based at the (institution blinded for journal review) sports center. All participants provided informed consent and no financial incentives were provided for study participation. Volunteers were only excluded if they had a history of previous knee injury or surgery, or a body mass index (BMI) > 25 kg/m^2^.

**Fig. 1:**
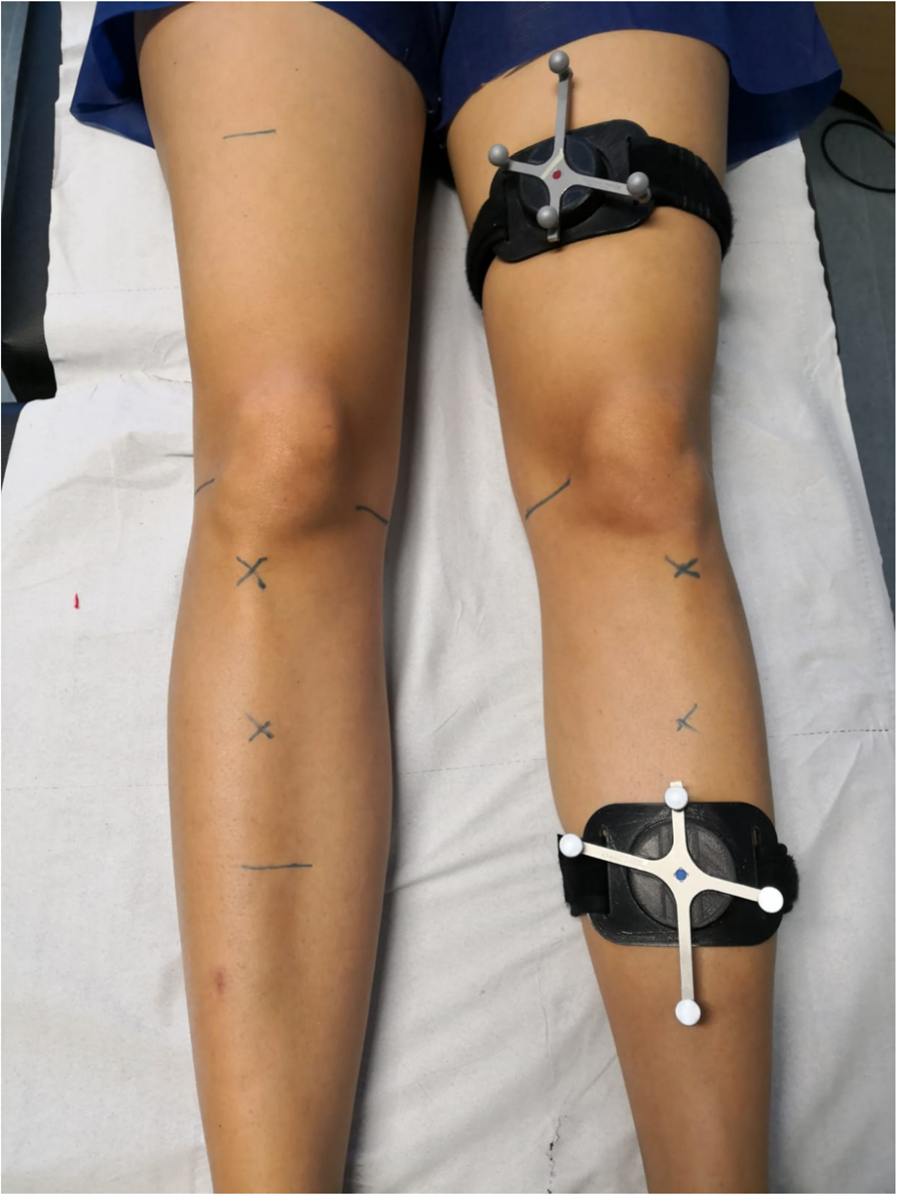
Photograph demonstrating the standardized placement of femoral and the tibial markers at 15 cm superior and 25 cm distal to the superior pole of the patella

All participants underwent evaluation of knee laxity parameters using a custom-made modification of the *OrthoPilot ACL navigation system* (version 2.0, B. Braun Aesculap, Tuttlingen, Germany) which allowed the reflective marker and transmitter arrays to be attached to the lower limbs of the athletes in a non-invasive manner using hypoallergenic custom-made elastic bandages and Velcro tape. The arrays were attached in a standardized manner at 15 cm proximal, and 25 cm distal to the upper pole of the patella (Fig. 1).

**Fig. 2:**
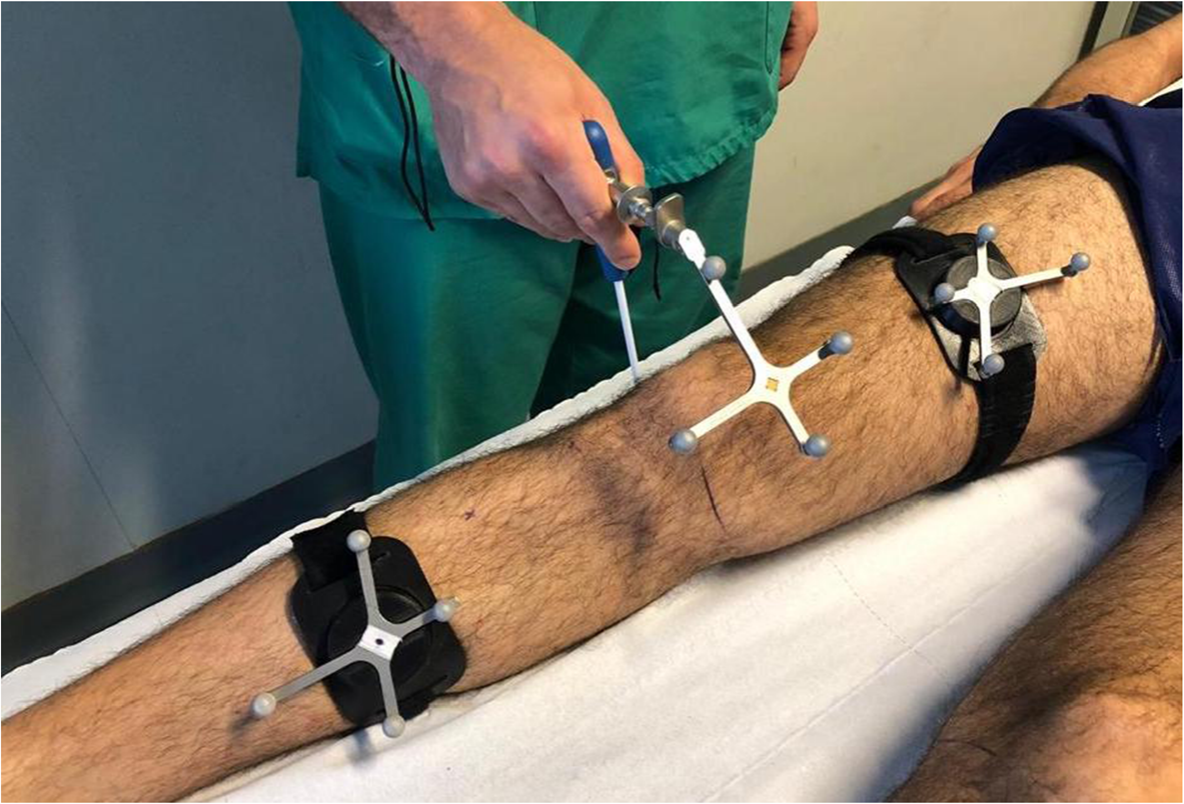
Photograph demonstrating the calibration procedure. This involves acquiring anatomic bony landmarks as requested by the software (tibial tuberosity, anterior crest of the tibia medial tibial plateau of the tibia lateral tibial plateau of the tibia). The calibration procedure proceeds with the static registration of the articular kinematics of the knee which consists of recording full extension of the knee at 0 degrees and then flexion of the knee at 90 degrees

Prior to any data acquisition, the volunteers were placed in a supine position on the examination table and a calibration process for the navigation system was undertaken. This involved placing the athletes’ dominant knee between 180 and 200 cm from the camera, and then using the navigation system to acquire anatomic bony landmarks requested by the software (tibial tuberosity, the anterior crest of the tibia and the medial and lateral points of the tibial plateau). Following this, static registrations of knee joint kinematics were recorded by placing the knee in full extension (0 degrees) and then at 90 degrees of flexion. (Fig. 2). Subsequently, registration of kinematics during passive range of motion between full extension and 90 degrees of flexion was undertaken. This completed the calibration process.

Two examiners independently performed physical examination of the dominant knee of each athlete. The main examiner was an experienced orthopaedic surgeon (consultant surgeon), the co-examiner was a fifth-year resident in orthopaedic surgery (resident surgeon). All participants underwent knee laxity examination on two separate occasions. At the first visit (time zero) each athlete was evaluated by the consultant and resident surgeons independently, and in a non-sequential manner. Six weeks after the first visit all the participants were re-tested only by the resident surgeon; the co-examiner was blinded to the outcomes of the previous physical examination.

The following knee laxity parameters were recorded during physical examination using the navigation software.

- Static evaluation
  - Anterior tibial translation (ATT) at 30° of flexion (Lachman maneuver) [26] expressed in millimeters (mm);
  - Passive total range of tibial rotation (ROT) at 30° of flexion, expressed in degrees. Rotation was calculated as the total range of rotation because the system was not able to calculate internal and external rotation separately.
- Dynamic evaluation
  - Anterior-posterior (AP) tibial translation and maximal tibial internal rotation (IR) during the PS maneuver expressed in millimeters and degrees, respectively. The PS maneuver was performed according to *Galway and MacIntosh* [8]; the knee was passively flexed from full extension to 40° - 50° of flexion with manual application of internal tibial torque and valgus stress with the hip abducted and flexed.

### Statistical analysis

All analyses were performed using *SPSS software* (version 25.0; SPSS, Chicago, Illinois, USA). Statistical significance was set at *p* < 0.05. The inter- and intra-observer reliability of all knee laxity parameters evaluated was calculated using the intraclass correlation coefficient (ICC) and evaluated according to the criteria of *Cicchetti* (*poor*; reliability coefficient is below 0.4, *fair;* between 0.4 and 0.59, *good;* between 0.6 and 0.74 is good, *excellent;* greater than 0.75) [6]. A fixed ICC model was used, in which both subjects and raters are assumed to be randomly selected from a wider pool of possible subjects and raters (i.e., the two-way random effect model) in order to evaluate absolute agreement.

## Results

One hundred volunteers were recruited to the study. Demographic details are reported in Table 1.

**Table 1:** Demographic details and characteristics of the study population

	Total *(n =* 100)
Age at inclusion, mean ± SD	25.5 ± 2.4
Gender: female, *n (%)*	38 (38)
BMI (kg/m^2^), mean ± SD	21.1 ± 5.3
Dominant side: left, n (%)	17 (17)
Type of sports participation, *n (%)*	
Running	36 (36)
Soccer	27 (27)
Volleyball	20 (20)
Rugby	9 (9)
Tennis	8 (8)

All volunteers underwent non-invasive knee laxity assessment and there were no complications related to the examination process.

Static evaluation: the mean static ATT at 30° of knee flexion (Lachman maneuver) at time zero was 10.7 mm (SD ± 1.5) and 11 mm (SD ± 1.4) for consultant and resident operators respectively; the mean static ROT at 30° of knee flexion at time zero was 31.1° (SD ± 4.8) and 31.4° (SD ± 4.5) for consultant and resident operators respectively.

**Fig. 3:**
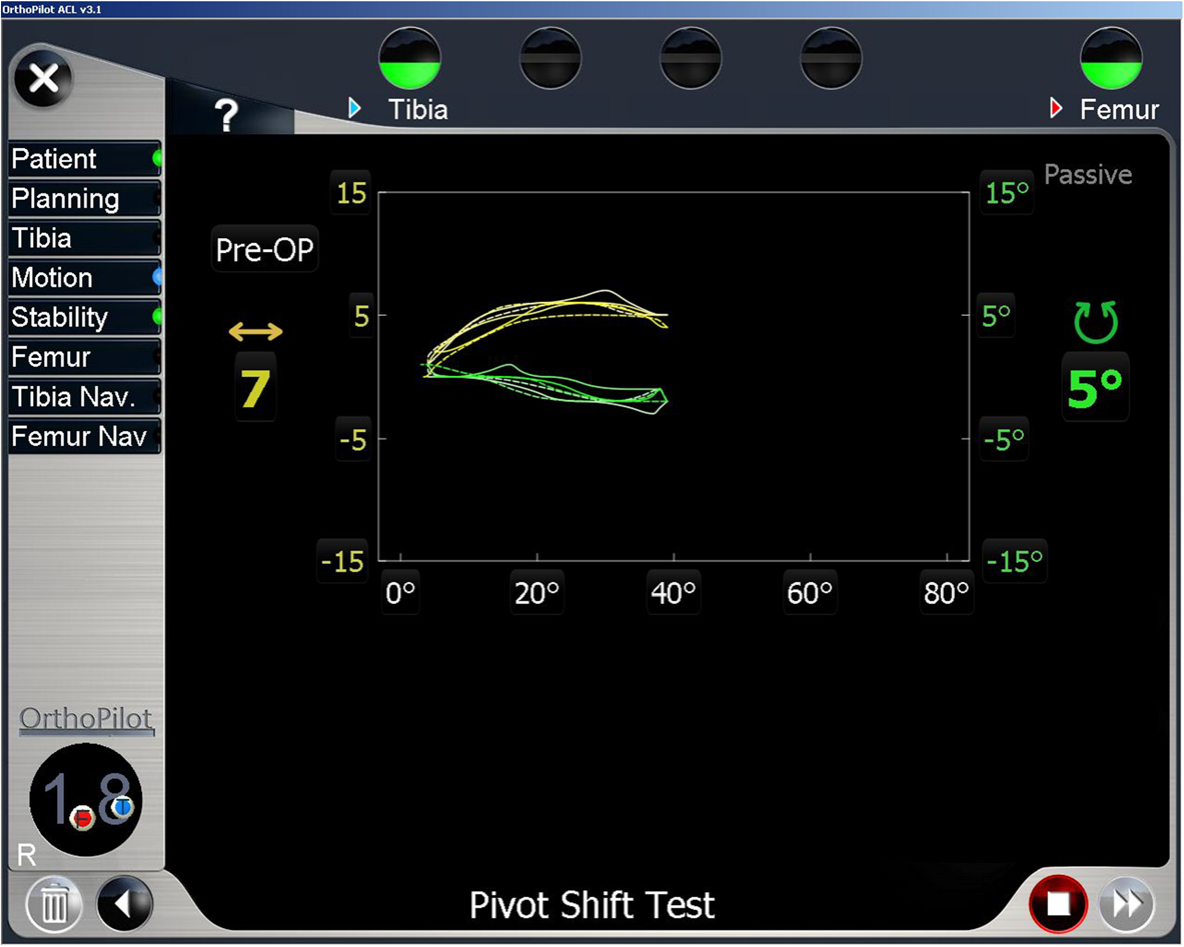
Quantitative pivot shift maneuver analysis using the non-invasive navigation system. The navigation screen during the pivot shift meneuver, with the X-axis representing the knee flexion angle, the left Y-axis representing tibial translation relative to the femur (+; anterior translation), and the right Y-axis representing tibial rotation relative to the femur (+; internal rotation). The yellow line represents tibial translation, and the green line represents tibial rotation

Dynamic evaluation: the mean dynamic AP tibial translation at time zero was 12.5 mm (SD ± 6.9) and 13.3 mm (SD = ± 6.9) for consultant and resident operators respectively; the mean dynamic maximal tibial IR during the PS maneuver at time zero was 12.9° (SD ± 4.9) and 12.7° (SD ± 4.5) for consultant and resident operators respectively. During the dynamic evaluation, the kinematic information was relayed to the computer and used by the software to display real-time images demonstrating the two components of the PS phenomenon. (Fig. 3) Knee laxity parameters and results of the static and dynamic navigated evaluations are reported in Table 2.

**Table 2:** Knee laxity parameters and results of the static and dynamic navigated evaluation

	Time zero			Retest	
*mean (± SD)*	*Senior*	*Resident*	*Range (v.min - v.max)*	*Resident*	*Range*
**Static anterior tibial translation (ATT)***(Lachman test); (mm)*	10.7 (± 1.5)	11 (± 1.4)	6–15	10.8 (± 1)	8–13
**Static total passive tibial rotation (ROT)***(degrees)*	31.1 (± 4.8)	31.4 (± 4.5)	17–48	31.7 (± 4.3)	22–45
**Dynamic AP tibial translation***(Pivot shift maneuver); (mm)*	12.5 (± 6.9)	13.3 (± 6.9)	3–35	12.4 (± 5.9)	5–34
**Dynamic maximal tibial IR***(Pivot shift maneuver); (degrees)*	12.9 (± 4.9)	12.7 (± 4.5)	3–29	11.4 (± 3.3)	5–22

**Fig. 4:**
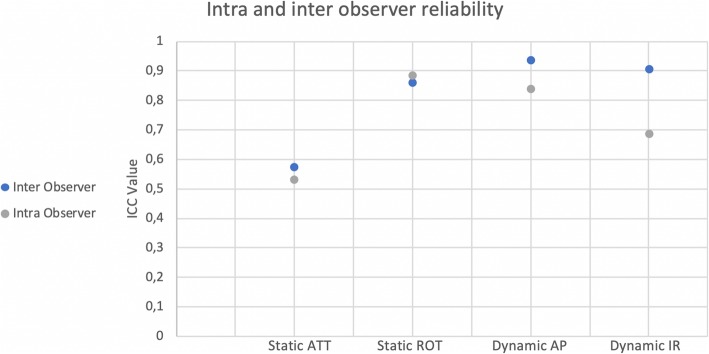
Scatter plot representing the distribution of the ICC, with intra and interclass reliability in Healthy Volunteers

The results of the evaluation of inter- and intra-observer reliability for each test are comprehensively reported in Table 3 and Fig. 4. In summary, the inter-observer correlation ranged from fair for ATT (0.572) to excellent for ROT (0.859), and for the dynamic components of the PS (0.936 for AP tibial translation, and 0.903 for IR). For intra-observer correlation, the ICC was fair for ATT (0.529), good for dynamic IR (0.684) and excellent for the other components analyzed (0.883 for ROT, and 0.837 for dynamic AP tibial translation).

**Table 3:** Results of intra- and interobserver reliability ICC

	Interclass correlation coefficient	95% Confidence interval		
		Lower Bound	Upper Bound	Sig.
Static anterior tibial translation (ATT) (mm); (Lachman test) Inter Observer	**0,572**	0,366	0,712	< 0,001
Static anterior tibial translation (ATT) (mm); (Lachman test) Intra Observer	**0,529**	0,304	0,682	< 0,001
Static total passive tibial passive rotation (ROT) (Degrees) Inter Observer	**0,859**	0,791	0,905	< 0,001
Static total passive tibial passive rotation (ROT) (Degrees) Intra Observer	**0,883**	0,826	0,921	< 0,001
Dynamic AP tibial translation (mm); (Pivot shift maneuver) Inter Observer	**0,936**	0,904	0,958	< 0,001
Dynamic AP tibial translation (mm); (Pivot shift maneuver) Intra Observer	**0,837**	0,756	0,89	< 0,001
Dynamic maximal tibial IR (degrees); (Pivot shift maneuver) Inter Observer	**0,903**	0,856	0,935	< 0,001
Dynamic maximal tibial IR (degrees); (Pivot shift maneuver) Intra Observer	**0,684**	0,517	0,792	< 0,001

*When the reliability coefficient is below 0.4, the level of significance is poor; when is between 0.4 and 0.59 the level of significance is fair; when it is between 0.6 and 0.74 is good; when is over 0.75 is excellent.*

## Discussion

The main finding of this study was that the use of non-invasive navigation for the assessment of knee laxity in healthy volunteers is associated with fair to excellent inter- and intra-observer reliability. This is an important finding because to our knowledge, although the use of this technology has previously been reported, the inter- and intra-observer reliability in awake subjects has not.

In a recent study investigating the use of non-invasive navigation to evaluate the PS, *Maeda* et al. [21] demonstrated moderate correlation with the clinical grade of PS, and also with measurements obtained using commercial transmitters fixed to the bone at the time of ACL reconstruction. In conjunction with the current study, these findings suggest that non-invasive navigation could be a potentially useful modality in the clinical assessment of knee laxity. However, the current study recruited a population of healthy volunteers. The rationale for this was based upon the ability to rapidly recruit a large study population in order to undertake a “proof of concept” evaluation prior to embarking on a clinical study. It is the opinion of the authors that the findings of this study support this technology and justify that further validation is performed with ACL-injured patients.

Although the PS is a cornerstone of knee laxity assessment, it has several major limitations. There is a clear need for further study on this topic because it is well recognized that evaluation of knee laxity parameters in contemporary practice remains very subjective. This subjectivity is a major factor contributing to the broad ranges of inter- and intra-observer reliability reported for PS evaluation, and this limits the importance that can be attributed to the data obtained. Recently, numerous devices have been developed and evaluated in order to try to quantify the PS test more objectively [27]. This has included navigation systems [14, 17], electromagnetic systems [5, 12, 13, 22], accelerometers [10, 18, 20] and image analysis devices [11, 23]. However, in a recent systematic review Grassi et al. [9] concluded that no “gold standard” methodology for the evaluation of the pivot shift has been established. For this reason the publication of inter- and intra-observer reliabilities is likely to be useful in aiding comparisons between these technologies and defining their roles in clinical practice.

Rotatory knee instability in the ACL-deficient knee is an abnormal, complex three-dimensional motion comprised of translation and rotation of the tibia along its axis and is commonly called “antero lateral rotatory instability” (ALRI). ALRI can be evaluated by manual testing consisting mainly of the PS test, which was firstly described by *Slocum and Larson* in 1968 [26]*.* More recently, *Bull and Amis* described the PS phenomenon as a sudden rotation of the tibia relative to the femur occurring in an ACL-injured knee under a valgus torque at low angles of knee flexion [2]. They also stated that the PS phenomenon is composed of both rotation of the tibia about its long axis, and a translational component related to the anterior subluxation of the lateral tibial plateau, followed by its sudden reduction [2–4]*.* The navigation system used in the present study allowed a real time calculation of these two components of the PS phenomenon.

Although conventional navigation (with osseous fixation) is well validated in the evaluation of the PS test [10, 16, 24] the use of non-invasive navigation requires independent validation because of some potential limitations. This includes the possible movement between the surface transmitters and the skin, and also between the transmitters and the osseous anatomy of the subjects evaluated. Despite these concerns, the results of the current study demonstrate good to excellent inter- and intra-observer reliability of the system for the evaluation of the pivot shift maneuver suggesting that the degree of error is likely small and that non-invasive navigation is a potentially useful tool for objective quantification of rotational and translation kinematics of the tibia during the PS test. For static evaluation of ATT (Lachman test) the ICC was only fair, but it should be considered that the accuracy of the navigation system is ±1 mm, and in 88% of the tests the difference between observations was within ±2 mm. Possible explanations of the fair agreement in static ATT evaluation (Lachman test) are multifactorial and include accuracy of the system, skin motion artefacts rather and differences in technique between operators. Despite that, the findings are promising, but it is important to note that this was a preliminary study on healthy subjects. Therefore, further studies are needed to evaluate whether data acquired from non-invasive navigation correlates with the clinical grade of PS in ACL-injured patients.

Although the non-invasive navigation system demonstrated fair to excellent reliability it is also important to note its disadvantages when compared to other technologies intended for the purpose of objective measurement of the PS. The main disadvantage is the need for specialist equipment and its associated costs, lack of portability and the space required for storage, making them relatively impractical in the office or pitch-side setting [7, 10]*.* Clearly there is a need to improve the technology in order to include cameras and software in a portable device such as a computer or a laptop.

The main limitation of this study was that a comparison with standard navigation techniques (using bony fixation) was not possible. Furthermore, the evaluation was performed in awake volunteers. It is recognized that PS evaluation is more reliable in patients under general anaesthesia [7, 11]*.* However, it is also the case that the PS test is routinely performed without anaesthesia in daily practice, and so it was important to determine the ICC in this scenario. An additional limitation is that the study participants were healthy subjects with normal knee kinematics, and this could theoretically lead to higher Inter- and intra-observer reliabilities than if injured knees with abnormal kinematics had been studied. A further limitation was that no correlation was undertaken between the clinical grade of PS (typically graded according to the IKDC classification) and knee laxity data acquired with non-invasive navigation. Therefore, the results of this study cannot be extrapolated to the evaluation and grading of the pivot shift test in ACL injured knees. However, the results of this study demonstrate fair to excellent reliability and this provides justification for a clinical study.

## Conclusion

Non-invasive navigation for the assessment of knee laxity is associated with fair to excellent inter- and intra-observer reliability in a population of healthy volunteers with normal knee kinematics.

## Data Availability

The datasets generated during and/or analyzed during the current study are not publicly available but are available from the corresponding author on reasonable request.

## Abbreviations

ACL: Anterior cruciate ligament
ALRI: Antero lateral rotational instability
AP: Anterior posterior
ATT: Anterior tibial translation
BMI: Body mass index
DoF: Degrees of freedom
ICC: Intraclass correlation coefficient
IKDC: International Knee Documentation Committee
IR: Internal rotation
PS: Pivot shift
ROM: Range of motion
ROT: Passive tibial rotation

## References

[1] Arilla FV, Rahnemai-Azar AA, Yacuzzi C, Guenther D, Engel BS, Fu FH et al (2016) Correlation between a 2D simple image analysis method and 3D bony motion during the pivot shift test. Knee. 10.1016/j.knee.2016.06.003 10.1016/j.knee.2016.06.003 27810428

[2] Bull AMJ, Amis AA (1998) The pivot-shift phenomenon: a clinical and biomechanical perspective. Knee. 10.1016/S0968-0160(97)10027-8

[3] Bull AMJ, Amis AA (1998) Knee joint motion: description and measurement. Proc Inst Mech Eng Part H J Eng Med. 10.1243/0954411981534132 10.1243/0954411981534132 9803155

[4] Bull AMJ, Andersen HN, Basso O, Targett J, Amis AA (1999). Incidence and mechanism of the pivot shift: an in vitro study. Clin Orthop Relat Res.

[5] Bull AMJ, Berkshire FH, Amis AA (1998) Accuracy of an electromagnetic measurement device and application to the measurement and description of knee joint motion. Proc Inst Mech Eng Part H J Eng Med. 10.1243/0954411981534123 10.1243/0954411981534123 9803154

[6] Cicchetti DV (1994) Guidelines, criteria, and rules of thumb for evaluating normed and standardized assessment instruments in psychology. Psychol Assess. 10.1037/1040-3590.6.4.284

[7] Colombet P, Jenny JY, Menetrey J, Plaweski S, Zaffagnini S (2012) Current concept in rotational laxity control and evaluation in ACL reconstruction. Orthop Traumatol Surg Res doi. 10.1016/j.otsr.2012.10.005 10.1016/j.otsr.2012.10.005 23153665

[8] Galway HR, MacIntosh DL (1980) The lateral pivot shift: a symptom and sign of anterior cruciate ligament insufficiency. Clin Orthop Relat Res. 10.1097/00003086-198003000-00008 7371314

[9] Grassi A, Lopomo NF, Rao AM, Abuharfiel AN, Zaffagnini S (2016) No proof for the best instrumented device to grade the pivot shift test: a systematic review. J ISAKOS Jt Disord Orthop Sport Med. 10.1136/jisakos-2015-000047

[10] Helfer L, Vieira TD, Praz C, Fayard JM, Thaunat M, Saithna A et al (2019) Triaxial accelerometer evaluation is correlated with IKDC grade of pivot shift. Knee Surgery, Sport Traumatol Arthrosc. 10.1007/s00167-019-05563-7 10.1007/s00167-019-05563-7 31201443

[11] Hoshino Y, Araujo P, Irrgang JJ, Fu FH, Musahl V (2012) An image analysis method to quantify the lateral pivot shift test. Knee Surgery, Sport Traumatol Arthrosc. 10.1007/s00167-011-1845-x 10.1007/s00167-011-1845-x PMC3309139 22203048

[12] Hoshino Y, Kuroda R, Nagamune K, Araki D, Kubo S, Yamaguchi M et al (2012) Optimal measurement of clinical rotational test for evaluating anterior cruciate ligament insufficiency. Knee Surgery, Sport Traumatol Arthrosc. 10.1007/s00167-011-1643-5 10.1007/s00167-011-1643-5 21850429

[13] Hoshino Y, Kuroda R, Nagamune K, Yagi M, Mizuno K, Yamaguchi M et al (2007) In vivo measurement of the pivot-shift test in the anterior cruciate ligament-deficient knee using an electromagnetic device. Am J Sports Med. 10.1177/0363546507299447 10.1177/0363546507299447 17351123

[14] Ishibashi Y, Tsuda E, Yamamoto Y, Tsukada H, Toh S (2009) Navigation evaluation of the pivot-shift phenomenon during double-bundle anterior cruciate ligament reconstruction: is the posterolateral bundle more important? Arthrosc - J Arthrosc Relat Surg. 10.1016/j.arthro.2008.10.008 10.1016/j.arthro.2008.10.008 19409306

[15] Kim SJ, Kim HK (1995). Reliability of the anterior drawer test, the pivot shift test, and the Lachman test. Clin Orthop Relat Res.

[16] Koh J (2005) Computer-assisted navigation and anterior cruciate ligament reconstruction: accuracy and outcomes. Orthopedics. 10.3928/0147-7447-20051002-16 10.3928/0147-7447-20051002-16 16235456

[17] Lane CG, Warren RF, Stanford FC, Kendoff D, Pearle AD (2008) In vivo analysis of the pivot shift phenomenon during computer navigated ACL reconstruction. Knee Surgery, Sport Traumatol Arthrosc. 10.1007/s00167-008-0504-3 10.1007/s00167-008-0504-3 18311485

[18] Lopomo N, Signorelli C, Bonanzinga T, Muccioli GMM, Visani A, Zaffagnini S (2012) Quantitative assessment of pivot-shift using inertial sensors. Knee Surgery, Sport Traumatol Arthrosc. 10.1007/s00167-011-1865-6 10.1007/s00167-011-1865-6 22222615

[19] Lopomo N, Zaffagnini S, Bignozzi S, Visani A, Marcacci M (2010) Pivot-shift test: analysis and quantification of knee laxity parameters using a navigation system. J Orthop Res. 10.1002/jor.20966 10.1002/jor.20966 19642114

[20] Lopomo N, Zaffagnini S, Signorelli C, Bignozzi S, Giordano G, Marcheggiani Muccioli GM et al (2012) An original clinical methodology for non-invasive assessment of pivot-shift test. Comput Methods Biomech Biomed Engin. 10.1080/10255842.2011.591788 10.1080/10255842.2011.591788 21728739

[21] Maeda S, Tsuda E, Yamamoto Y, Naraoka T, Kimura Y, Ishibashi Y (2016) Quantification of the pivot-shift test using a navigation system with non-invasive surface markers. Knee Surgery, Sport Traumatol Arthrosc. 10.1007/s00167-016-4165-3 10.1007/s00167-016-4165-3 27306984

[22] Milne AD, Chess DG, Johnson JA, King G (1996) Accuracy of an electromagnetic tracking device: a study of the optimal operating range and metal interference. J Biomech. 10.1016/0021-9290(96)83335-5 10.1016/0021-9290(96)83335-5 9147976

[23] Muller B, Hofbauer M, Rahnemai-Azar AA, Wolf M, Araki D, Hoshino Y et al (2016) Development of computer tablet software for clinical quantification of lateral knee compartment translation during the pivot shift test. Comput Methods Biomech Biomed Engin. 10.1080/10255842.2015.1006210 10.1080/10255842.2015.1006210 25659403

[24] Musahl V, Griffith C, Irrgang JJ, Hoshino Y, Kuroda R, Lopomo N et al (2016) Validation of quantitative measures of rotatory knee laxity. Am J Sports Med. 10.1177/0363546516650667 10.1177/0363546516650667 27371547

[25] Noyes FR, Grood ES, Cummings JF, Wroble RR (1991). An analysis of the pivot shift phenomenon. The knee motions and subluxations induced by different examiners. Am J Sports Med.

[26] Slocum DB, Larson RL (1968) Rotatory instability of the knee. Its pathogenesis and a clinical test to demonstrate its presence. J Bone Joint Surg Am. 10.2106/00004623-196850020-00001 5642814

[27] Sundemo D, Alentorn-Geli E, Hoshino Y, Musahl V, Karlsson J, Samuelsson K (2016) Objective measures on knee instability: dynamic tests: a review of devices for assessment of dynamic knee laxity through utilization of the pivot shift test. Curr Rev Musculoskelet Med doi. 10.1007/s12178-016-9338-7 10.1007/s12178-016-9338-7 PMC4896881 26984465

